# Evaluation of cervical cancer detection with acoustic radiation force impulse ultrasound imaging

**DOI:** 10.3892/etm.2013.1057

**Published:** 2013-04-09

**Authors:** YIJIN SU, LIANFANG DU, YING WU, JUAN ZHANG, XUEMEI ZHANG, XIAO JIA, YINGYU CAI, YUNHUA LI, JING ZHAO, QIAN LIU

**Affiliations:** 1 Departments of Ultrasound, Shanghai First People’s Hospital, School of Medicine, Shanghai Jiaotong University, Shanghai 200080, P.R. China; 2 Obstetrics and Gynecology, Shanghai First People’s Hospital, School of Medicine, Shanghai Jiaotong University, Shanghai 200080, P.R. China; 3 Pathology, Shanghai First People’s Hospital, School of Medicine, Shanghai Jiaotong University, Shanghai 200080, P.R. China

**Keywords:** acoustic radiation force impulse, cervical cancer, diagnosis

## Abstract

The aim of this study was to evaluate the application of acoustic radiation force impulse (ARFI) ultrasound imaging and its potential value in the characterization of cervical cancer. ARFI ultrasound imaging of the uterine cervix was performed in 58 patients with cervical cancer prior to surgery. The diagnosis of cervical cancer was confirmed by pathological results in each case. eSie Touch elastography imaging (EI), Virtual Touch tissue imaging (VTI) and Virtual Touch tissue quantification (VTQ; Siemens Medical Solutions, Mountain View, CA, USA) were used to qualitatively and quantitatively analyze the elasticity and hardness of lesions. For statistical analysis, the non-parametric Mann-Whitney U test and the Student’s u test were used to compare the elastic parameters and the results. EI showed that, compared with the surrounding cervical tissue, 72.41% (42 of 58) of the malignant lesions showed 4th or 5th grade images and 27.59% (16 of 58) had 3rd grade images. The EI images showed a significant difference between the malignant lesions and the surrounding normal tissues (P<0.001). VTI showed that compared with the surrounding cervical tissue, 84.48% (49 of 58) of the malignant lesions were stiffer than the surrounding tissues and 15.52% (9 of 58) had black and white honeycomb-like images. The VTI images showed a significant difference between the malignant lesions and normal cervical tissues (P<0.001). The surrounding normal tissues had lower VTQ values, with a mean of 2.11±1.19 m/sec, while the VTQ values in malignant lesions were higher than the surrounding normal tissues (3.41±1.59 m/sec, P<0.001). ARFI ultrasound imaging of the uterine cervix may be an objective method for the assessment of soft tissues. It has high sensitivity and specificity in the evaluation of cervical cancer and therefore has good diagnostic value in clinical applications.

## Introduction

Cervical cancer is the most common carcinoma of the genital tract in women, with an age-standardized incidence of 8.9 cases per 100,000 women/year (1), and an estimated 150,000 new cases per year in China (2), particularly in young women.

The diagnosis of cervical cancer now relies on specialized clinical examinations, computed tomography (CT), magnetic resonance imaging (MRI) and ultrasound. Compared with CT, which has a low contrast resolution of soft tissue, MRI is the ideal modality for visualization of the cervix (3). However, it is not usually possible to perform MRI immediately due to inconvenience and the limitations of contraceptive devices. By contrast, ultrasound is gaining clinical interest since it is less time consuming, cheaper, noninvasive and safe, particularly for patients undergoing repeated examination.

Acoustic radiation force impulse (ARFI) ultrasound imaging, as a new technique of elastography, works on the principle that different tissues have different coefficients of elasticity and therefore are under different levels of strain. Color coding and relative shear wave velocity (SWV) have been demonstrated with ARFI ultrasound imaging. According to the elasticity of pathological tissues, it is possible to conclude qualitatively and quantitatively the benign or malignant pathological tissue (4–8). However, ARFI ultrasound imaging uses a short-duration acoustic push pulse technique and is a non-manual technique, other elastic techniques require manual vibration. (9–11).

ARFI, a new technique of ultrasonic elastography, as a recent development of imaging technology, is able to obtain qualitative and quantitative information of the elasticity distribution within tissues and has the advantages of being noninvasive, painless and convenient. Hence, it has significant clinical value and wide application prospects. Currently ARFI ultrasound imaging is used for evaluation of tissue elasticity in the liver, pancreas, breast, thyroid and prostate (12–19). However, there are few reports concerning its use in cervical cancer (20). It is reported that ARFI has been used in transvaginal ultrasound imaging (21). However, although transvaginal ultrasound has numerous advantages, it is not applied to women that have not had sexual intercourse, it may cause bleeding and infection and a number of women will not accept this examination method (22). Transvaginal ultrasound does not reveal the field in which cervical cancer occurs, including metastases to the pelvic cavity, the larger lymph nodes and violation of bladder and rectum. Therefore we suggest the use of transabdominal ultrasound with ARFI ultrasound imaging for the evaluation of cervical cancer. ARFI ultrasound imaging may be applied to all women, is safe, painless, convenient and likely to be accepted by patients. In addition, it is possible to identify more information about cervical cancer and the surrounding tissues, particularly distant metastases. This is important for the physician when determining the area of infiltration and planning treatment. The current study was designed to investigate the clinical value of ARFI ultrasound imaging in the prediction of cervical malignancies by detecting changes in tissue stiffness.

## Materials and methods

### General information

Fifty-eight consecutive patients were selected from the Department of Obstetrics and Gynecology of the Shanghai First People’s Hospital, School of Medicine, Shanghai Jiaotong University between March 2012 and October 2012. The inclusion criterion was the presence of lesions in the uterine cervix with definite pathological results. The exclusion criteria were a lesion diameter of <5 mm and a depth between the lesion and skin of >80 mm. To avoid infection and vaginal bleeding, and to enable the examination of women that have not had sexual intercourse, we used a transabdominal rather than transvaginal sonographic probe. A total of 58 women were enrolled (mean age 53.6±18.9 years, range 22–78 years) in the study. This study was conducted in accordance with the Declaration of Helsinki and with approval from the Ethics Committee of Shanghai Jiaotong University (Shanghai, China). Written informed consent was obtained from all participants.

### Acquisition of the ARFI data

Real-time ARFI ultrasound imaging was performed using an Acuson S2000 diagnostic ultrasound system (Siemens Healthcare, Erlangen, Germany) equipped with a 3.5 MHz abdominal probe. All examinations were performed in succession by two independent sonographers. The sonographers had >10 years experience in ultrasonic scanning. They were blinded to the colposcopy findings and physical examination results when performing the examinations.

The patients were asked to lie in a supine position with a half-full bladder. Conventional sonography was used to observe the shape, size, boundary and echoes of each lesion. Color Doppler was used to access the blood supply of the lesions. The highest sensitivity for detection of color Doppler signals was used, allowing for the detection of blood flow velocities ≥2 cm/sec. Using the ARFI elasticity model, elastography imaging (EI), Virtual Touch tissue imaging (VTI) and Virtual Touch tissue quantification (VTQ) were used to measure the elasticity of the lesions and surrounding tissues in turn.

### eSie Touch EI

The eSie Touch EI method generates grayscale and color scale elastograms. On the grayscale image, with increasing stiffness of the tissue, the images gradually change from white to black. White depicts the softest tissue and black, the hardest tissue. On the color scale, with increasing stiffness of the tissue, the image gradually changes from red to blue. Red represents the softest tissue and blue the hardest tissue (23).

According to Thomas *et al*(24), analysis of hardness classification of EI was as follows: 1st grade: definitely normal: 2/3 area was green, 1/3 was red, blue was negligible; 2nd grade: approximately normal, 2/3 area was green, 1/3 area was red and blue; 3rd grade: between normal and abnormal, 2/3 area was green, 1/3 area was red and blue; 4th grade: abnormal, blue area in the cervix was more extensive than red area, and blue area may extend beyond the cervix; 5th grade: definitely abnormal, blue area more extensive than red area, blue area markedly extends beyond the cervix.

### Virtual Touch tissue imaging

In the VTI model, the region of interest (ROI) should encircle the lesion. To obtain appropriate images, the probe was applied with light pressure to make complete contact with the abdomen. The VTI button was then pressed, a short (∼100 *μ*sec) acoustic push pulse was transmitted through tissue, and a black-and-white VTI image was obtained. A very stiff tissue may displace little or not at all. On the VTI elastic image, the softness or hardness of lesions and peripheral cervical tissue may be observed (25).

On the basis of the VTI classification method of Shuang-Ming *et al*(26) and research on thyroid nodule imaging, the images were divided into 4 types: ‘softer’, with the nodule whiter than the surrounding thyroid tissue; ‘equal stiffness’, with similar image colors of the nodule and the peripheral thyroid tissue; ‘stiffer’, with the nodule appearing blacker (>50%) than the surrounding thyroid tissue; and ‘cellular sample’, with the nodule showing an alternating black and white honeycomb-like distribution.

### Virtual Touch tissue quantification

In the VTQ model, the ROI (6×10 mm) was placed inside the lesion and the depth of the ROI was <80 mm. For more accurate and objectively derived elastic parameters, the ROI was placed inside lesions whose smallest diameters were >5 mm; it was continuously measured three times randomly, and the average value was calculated as the VTQ value (m/sec). The measurements of the surrounding tissues were performed with the ROI placed at the same level as the lesion and within 5–10 mm from the lesion, avoiding vascular structures; three consecutive elastic parameters were obtained and the average value was calculated as the VTQ value of the surrounding tissues.

### Statistical analysis

All statistical analysis used SPSS version 17.0 software (SPSS Inc, Chicago, IL, USA). All measured data were presented as the mean ± standard deviation. The EI and VTI image analysis used the non-parametric Mann-Whitney U test. Groups of lesions and peripheral normal tissue were compared using the Student U test. The χ^2^ test was used to calculate the sensitivity, specificity, positive predictive value, negative predictive value and diagnostic accordance rate.

## Results

### Lesion properties

From the 58 malignant lesions, 47 (81.03%) were squamous and 11 (18.97%) were adenocarcinoma. The lesion sizes ranged from 10×10 mm to 24×33 mm, with an average of 18.6×15.4 mm.

### Conventional sonography

The 58 malignant lesions all appeared solid on B-mode sonography, and all were hypoechoic. According to their morphologic characteristics, boundary, echoes on gray scale sonography and color Doppler flow imaging, the sensitivity, specificity and diagnostic accuracy were 78.95, 77.97 and 78.45%, respectively (Table I).

### eSie Touch elastography imaging analysis

**Figure 1:**
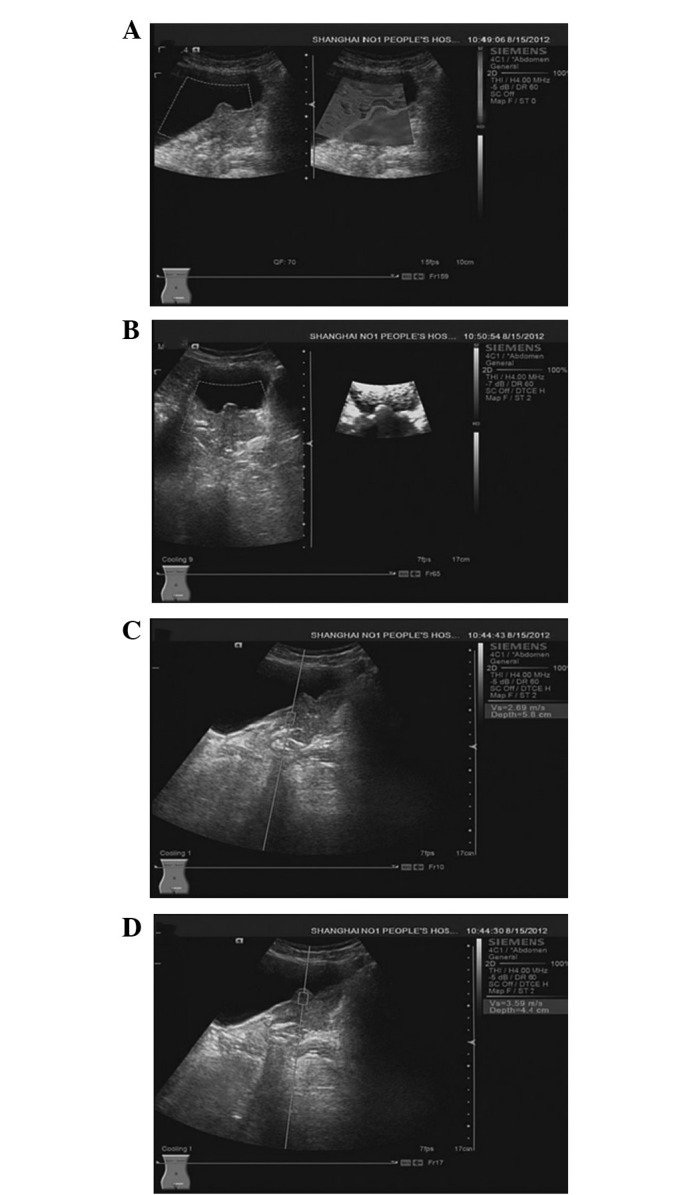
(A) EI of cervical cancer is markedly black at the area of cervical cancer; (B) VTI of cervical cancer shows a gray area at the site of the cervical cancer; (C) VTQ of surrounding normal tissue was 2.69 m/sec; (D) VTQ of cervical cancer was 3.59 m/sec. EI, elastography imaging; VTI, Virtual Touch tissue imaging; VTQ, Virtual Touch tissue quantification.

Compared with the surrounding cervical tissue, 72.41% (42 of 58) of the malignant lesions showed 4th or 5th grade images and 27.59% (16 of 58) had 3rd grade images (Table II; Fig. 1A). The EI images showed a significant difference between the malignant lesions and surrounding normal tissues (P<0.001).

### Virtual Touch tissue imaging analysis

Compared with the surrounding cervical tissue, 84.48% (49 of 58) of the malignant lesions showed stiffer images and 15.52% (9 of 58) had black and white honeycomb-like images (Table III; Fig. 1B). The VTI images showed a significant difference between malignant lesions and normal cervical tissues (P<0.001).

### Virtual Touch tissue quantification analysis

All the lesions were assessed at least three times by two independent observers based on various static images and the average value was recorded as the final result. The observers were blinded to the physical and pathological results. The assessments of the two observers had high consistency (κ=0.71).

Surrounding normal tissues had lower VTQ values (Table IV; Fig. 1C), with a mean of 2.11±1.19 m/sec, while the VTQ values in malignant lesions were higher than the surrounding normal tissues (3.41±1.59 m/sec, P<0.001; Fig. 1D).

## Discussion

Sonoelastography remains a primary method for the diagnosis of cervical cancer. Compared with cervical biopsy, which is the gold standard, women are more likely to accept noninvasive examination. A previous study has shown that there is a statistical difference of elasticity between malignant and normal cervical tissue. The stiffer the object, the larger the elastic modulus. Malignant tissues are stiffer than benign tissues. Therefore the elastic modulus of the former is greater than that of the latter. Cervical tissues mainly comprise collagen fiber and a few muscle fibers. Although cervical tissues may undergo changes to elasticity under different physiological conditions, for example, the elasticity may be affected by pregnancy or the menstrual cycle, the normal elasticity of cervical tissues does not change with age (24).

ARFI ultrasound imaging is a convenient examination method. We used eSie Touch EI utilizing ARFI ultrasound technology to qualitatively diagnose cervical cancer. eSie Touch imaging forms the elastogram by computing relative tissue deformation globally and displaying the information within a user-defined ROI. This method uses grayscale and color coding to show the relative stiffness of the tissues. The more black or blue a tissue appears, the stiffer the tissue is. Our study showed that the malignant tissues were stiffer than the surrounding tissues using EI.

In the current study, VTI utilizing ARFI ultrasound technology was used to qualitatively diagnose cervical cancer. A Virtual Touch software image is a qualitative grayscale map of relative tissue stiffness (elastogram) for a user-defined ROI. This method uses a grayscale to demonstrate the relative stiffness of the tissues. The blacker a tissue appears, the stiffer the tissue is. Our study showed that the malignant tissues were stiffer than the surrounding tissues using VTI.

VTQ utilizing ARFI ultrasound technology was used to quantitatively diagnose cervical cancer. In VTQ, an acoustic push pulse is applied to the ROI to induce a shear-wave. The time between the generation of the shear-wave and detection of the peak is utilized to compute the SWV. Multiple measurements were taken throughout the chosen location and the mean was calculated. This numerical value was correlated with the stiffness of tissue within the ROI. We used ARFI ultrasound imaging to obtain the SWV between the malignant and normal tissues. The stiffer a tissue, the greater its SWV (27,28). The present study showed that the malignant tissue was stiffer than the surrounding tissues using VTQ.

In the current study, a transabdominal scan probe was used to avoid infection and vaginal bleeding, particularly in women that have not had sexual intercourse. This method is able to scan the tissues surrounding the cervix, and examine the lymph nodes and violation of the bladder and rectum. It was important in determining the stage of cervical cancer and is the subject of our next study. However, the transabdominal probe has certain limitations, such as when examining deeper tissues or when used on patients with high adiposity.

In conclusion, ARFI ultrasound imaging is a superior method for the examination of cervical cancer. ARFI ultra-sound imaging of the uterine cervix may be an objective method for the assessment of soft tissue. ARFI ultrasound imaging has a high sensitivity and specificity in the evaluation of cervical cancer and therefore has a good diagnostic value in clinical applications.

## Figures and Tables

**Table I:** Diagnosis of cervical cancer and surrounding normal tissue by conventional sonography.

	Sonographic diagnosis		
Parameter	Suspected malignant	Suspected normal tissue	Total
Malignant	51	13	58
Normal tissue	12	49	58
Sensitivity, %			78.95
Specificity, %			77.97
Accuracy, %			78.45

**Table II:** Elastography imaging analysis of malignant and normal tissues.

	Lesion type		
Grade	Malignant (n=58)	Normal tissue (n=58)	Total
1	0	28	28
2	0	20	20
3	3	7	10
4	19	3	22
5	36	0	36

**Table III:** Virtual Touch tissue imaging analysis of malignant and normal tissues.

	Lesion type		
Characteristic	Malignant (n=58)	Normal tissue (n=58)	Total
Softer	4	25	29
Equal	18	21	39
Stiffer	32	4	36
Honeycomb	4	8	12

**Table IV:** Virtual Touch tissue quantification (VTQ) imaging analysis of malignant and normal tissues.

Tissue type	n	VTQ value, m/s	P-value
Malignant	58	3.41±1.59	<0.001
Normal tissue	58	2.11±1.19	
